# Plasma membrane proteoglycans syndecan-2 and syndecan-4 engage with EGFR and RON kinase to sustain carcinoma cell cycle progression

**DOI:** 10.1016/j.jbc.2022.102029

**Published:** 2022-05-13

**Authors:** DeannaLee M. Beauvais, Scott E. Nelson, Kristin M. Adams, Noah A. Stueven, Oisun Jung, Alan C. Rapraeger

**Affiliations:** Department of Human Oncology, University of Wisconsin Carbone Cancer Center, University of Wisconsin-Madison, Madison, Wisconsin, USA

**Keywords:** Abl tyrosine kinase, breast cancer, cell cycle, epidermal growth factor receptor (EGFR), head and neck cancer, integrin, p38MAPK, syndecan, macrophage stimulating receptor (MST1R), Recepteur d’Origine Nantais (RON), DDR, DNA damage response, EdU, 5-ethynyl-2′-deoxyuridine, EGF, epidermal growth factor, EGFR, epidermal growth factor receptor, HNSCC, head and neck squamous cell carcinoma, HTEs, human tonsillar epithelial cells, HU, hydroxyurea, LN332, laminin-332, NOKs, normal oral keratinocytes, P38MAPK, p38 mitogen-activated protein kinase, PCNA, proliferating cell nuclear antigen, RON, Recepteur d’Origine Nantais, Sdc2, syndecan-2, Sdc4, syndecan-4, SSTN_EGFR_, synstatin_EGFR_, TNBC, triple-negative breast cancer

## Abstract

Epidermal growth factor receptor (EGFR) is a causal factor in carcinoma, yet many carcinoma patients are resistant to EGFR inhibitors. Potential insight into this resistance stems from prior work that showed EGFR in normal epithelial cells docks to the extracellular domain of the plasma membrane proteoglycan syndecan-4 (Sdc4) engaged with α3β1 and α6β4 integrins. We now report that this receptor complex is modified by the recruitment of syndecan-2 (Sdc2), the Recepteur d’Origine Nantais (RON) tyrosine kinase, and the cellular signaling mediator Abelson murine leukemia viral oncogene homolog 1 (ABL1) in triple-negative breast carcinoma and head and neck squamous cell carcinoma, where it contributes to EGFR kinase–independent proliferation. Treatment with a peptide mimetic of the EGFR docking site in the extracellular domain of Sdc4 (called SSTN_EGFR_) disrupts the entire complex and causes a rapid, global arrest of the cell cycle. Normal epithelial cells do not recruit these additional receptors to the adhesion mechanism and are not arrested by SSTN_EGFR_. Although EGFR docking with Sdc4 in the tumor cells is required, cell cycle progression does not depend on EGFR kinase. Instead, progression depends on RON kinase, activated by its incorporation into the complex. RON activates ABL1, which suppresses p38 mitogen-activated protein kinase and prevents a p38-mediated signal that would otherwise arrest the cell cycle. These findings add to the growing list of receptor tyrosine kinases that support tumorigenesis when activated by their association with syndecans at sites of matrix adhesion and identify new potential targets for cancer therapy.

It is well known that cooperative signaling between adhesion receptors and receptor tyrosine kinases regulates mechanosensing, cell migration, proliferation, and survival. This cooperative signaling often emanates from adhesion sites that incorporate receptor tyrosine kinases along with integrins, cadherins, or other adhesion receptors, leading to clustering and both ligand-dependent and ligand-independent activation of the kinases (1, 2, 3, 4). However, whereas there are numerous reports of integrins associating with receptor tyrosine kinases, the means by which these individual receptors are recognized and organized into signaling foci often remains obscure.

Syndecans contain docking sites in their extracellular domains that assemble partner receptors into signaling complexes (5, 6, 7). In addition to the syndecan, these receptor complexes typically consist of one or more integrins together with a receptor tyrosine kinase or phosphatase (5, 6, 7, 8, 9, 10, 11). Because the docking motifs in the syndecans are extracellular, peptide mimetics of these sites (called “synstatins”) can be used as tools to competitively disrupt receptor assembly and probe the importance of these specific receptor interactions. Such studies have shown that receptors organized by syndecans impact signaling critical for tumor cell migration/invasion, proliferation, and survival and/or tumor-induced angiogenesis (6, 7, 8, 9, 12).

Syndecan-4 (Sdc4) mediates the assembly of the epidermal growth factor receptor (EGFR) with the laminin-332 (LN332)–binding α6β4 and α3β1 integrins (7, 13). Whereas the α6β4 integrin engages the Sdc4 cytoplasmic domain, coupling of EGFR and the α3β1 integrin relies on a juxtamembrane site in the Sdc4 ectodomain (amino acids 87–131 in humans), which is blocked by a peptide mimetic now called “synstatin-EGFR” (SSTN_EGFR_) (13). This peptide blocks the epidermal growth factor (EGF)-stimulated migration of keratinocytes and mammary epithelial cells on LN332 that the cells deposit as they migrate (7, 13).

EGFR has been implicated in multiple human cancers, including carcinomas of the head and neck and breast (14, 15, 16). EGFR and its ligands (*e.g.*, EGF, transforming growth factor-alpha) are overexpressed in up to 90% of head and neck cancer patients (17, 18, 19), are further induced by standard of care external beam radiation and DNA damaging agents (20, 21, 22), and are strongly linked to tumor progression (23, 24). EGFR is also a causal agent in triple-negative breast carcinoma (TNBC), a highly malignant form that comprises 15% to 25% of breast cancers (25, 26). Nonetheless, EGFR inhibitors, including the EGFR-blocking antibody cetuximab or EGFR kinase inhibitors, have had disappointing outcomes in the clinic (17, 27), suggesting alternative mechanisms through which EGFR promotes or sustains the progression of these cancers. Accordingly, we subjected head and neck squamous cell carcinoma (HNSCC) and TNBC cells for treatment with SSTN_EGFR_ to probe the involvement of Sdc4 as a partner in EGFR signaling in these cancers. We find that SSTN_EGFR_ induces a rapid and global cell cycle arrest in HNSCC and TNBC cells, including an S-phase arrest; this is unusual because, although growth factor receptors may be required to enter S-phase, their signaling is not thought to be required once S-phase is begun. We also find that nontransformed oral or mammary epithelial cells are refractory to proliferation arrest by SSTN_EGFR_, making its inhibition highly specific for tumor cells. Furthermore, although EGFR is required in this regulatory mechanism, its kinase activity is not. Instead, cell cycle progression depends upon active recepteur d’origine nantais (RON) (also known as macrophage stimulating protein-1 receptor (MST1R) (28, 29, 30)) and the cytoplasmic kinase ABL1 (31), which, along with the Sdc4 homolog syndecan-2 (Sdc2) partners with Sdc4, EGFR, and the laminin-binding integrins specifically in the tumor cells. When these kinases are inactivated by their displacement from Sdc4 by SSTN_EGFR_, a corresponding increase in p38 mitogen-activated protein kinase (p38MAPK) occurs, presumably constitutively activated by metabolic, oncogenic, and/or genotoxic stress in the tumor cells but held in abeyance by signaling from the Sdc4 receptor complex. This suggests that an epithelial cell migration mechanism that relies on Sdc4, EGFR, and the α3β1 and α6β4 integrins adopts a dual role by incorporating Sdc2, RON, and ABL1 to sustain S-phase progression at times of cellular stress.

## Results

### SSTN_EGFR_ impairs carcinoma cell proliferation

**Figure 1:**
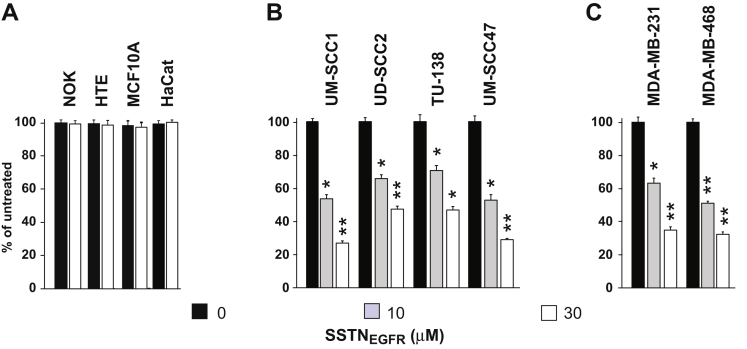
**Proliferation of nontransformed and transformed epithelial cells in SSTN**_**EGFR**_**.** Nontransformed oral keratinocytes (NOK), human tonsillar epithelia (HTE), breast (MCF10A) epithelial cells, and human epidermal keratinocytes (HaCat) (*A*), transformed UM-SCC1, UD-SCC2, TU-138, and UM-SCC47 HNSCC cells (*B*), and MDA-MB-231 and -468 TNBC (*C*) cells undergoing logarithmic proliferation are treated with 0, 10, or 30 μM SSTN_EGFR_ for 3 days, followed by quantification of cell number, expressed as a percentage of untreated (control) cells; ∗*p* ≤ 0.05, ∗∗*p* ≤ 0.01. HNSCC, head and neck squamous cell carcinoma; TNBC, triple-negative breast cancer.

The proliferation of a panel of human TNBC and HNSCC cell lines in the presence of SSTN_EGFR_ was compared to nontumorigenic breast, oral, and epidermal epithelial cells. Whereas the proliferation of nontumorigenic, immortalized normal oral keratinocytes (NOKs), human tonsillar epithelial cells (HTEs), mammary epithelial cells (MCF10A), and epidermal keratinocytes (HaCaT) are not affected by SSTN_EGFR_ (Fig. 1*A*), the peptide causes reduced proliferation of HNSCC (Fig. 1*B*) and TNBC cells (Fig. 1*C*) with an IC_50_ that falls with the range of 3 to 10 μM. This coincides with the approximate concentration required to displace 90% of EGFR and the α3β1 integrin from Sdc4 (13).

### SSTN_EGFR_ induces rapid cell cycle arrest

**Figure 2:**
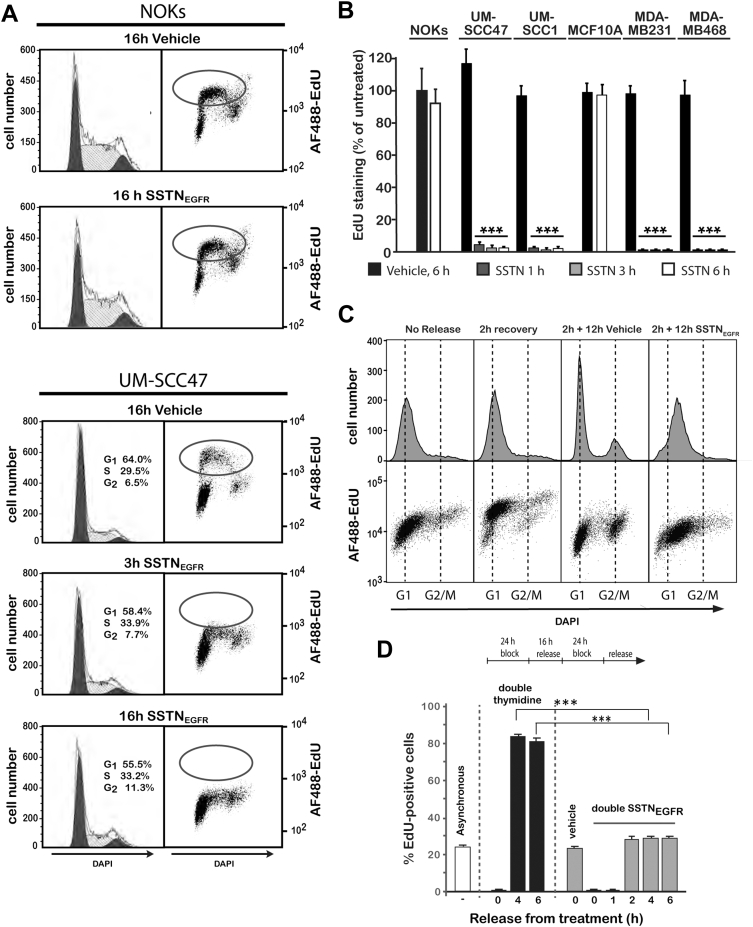
**Analysis of DNA synthesis and cell cycle progression in SSTN**_**EGFR**_**.***A*, NOK or UM-SCC47 cells were treated with or without 30 μM SSTN_EGFR_ for 3 h or 16 h, labeled with EdU and DAPI and analyzed by flow cytometry; *B*, transformed (UM-SCC47, UM-SCC1, MDA-MB-231, and MD-MB-468) and nontransformed (NOKs and MCF10A) epithelial cells were cultured with or without 30 μM SSTN_EGFR_ for 1, 3, or 6 h and then labeled with EdU. EdU incorporation is expressed as a percentage of NOKs grown in vehicle alone; *C*, UM-SCC47 cells were arrested at the G1/S phase interface using a double thymidine block (24 h block, 16 h release, and 24 h block), released for 2 h then subjected to 12 h treatment with either 30 μM SSTN_EGFR_ or vehicle alone. Cells were then labeled with EdU and DAPI and analyzed by flow cytometry; *D*, UM-SCC47 cells are subjected to a double thymidine or double SSTN_EGFR_ block (24 h block, 16 h release, and 24 h block), then released and labeled with EdU to quantify cells in S-phase; ∗∗∗*p* ≤ 0.001. EdU, 5-ethynyl-2′-deoxyuridine; NOK, normal oral keratinocyte.

For the sake of simplicity, we elected to use a single cell line (UWSCC47 cells) to conduct exploratory studies into this growth mechanism and then extended our findings to the other transformed cells. UM-SCC47 cells and normal NOKs as a control were treated with SSTN_EGFR_, accompanied by labeling with the thymidine nucleoside analog 5-ethynyl-2′-deoxyuridine (EdU), to analyze DNA synthesis by flow cytometry. As expected, treated NOKs showed no significant change in EdU incorporation or in the distribution of cells throughout the cell cycle when compared to untreated cells (Fig. 2*A*). In contrast, an S-phase block after only 3 h treatment of UM-SCC47 cells is indicated by a complete lack of EdU incorporation (Fig. 2*A*), and the failure of the cells to show any major change in their distribution in the G1-, S-, G2-, and M-phases of the cell cycle suggests the peptide may induce a global block to cell cycle progression. Shorter treatment times utilizing *in situ* staining of fixed cells show that treatment with SSTN_EGFR_ for as little as 1 h reduces EdU incorporation by well over 90% in HNSCC and TNBC cells, whereas NOKs and MCF10A cells show no reduction (Fig. 2*B*). To verify the arrest in S-phase, UM-SCC47 cells were synchronized in early S-phase using a double thymidine block, then released for 2 h to recover (Fig. 2*C*). Cells released for 2 h and chased for an additional 12 h with the vehicle alone progress into late S-phase/G2 or pass through G2/M and reenter G1 (Fig. 2*C*). In contrast, cells released for 2 h then chased for 12 h in SSTN_EGFR_ remain entirely in S-phase and show little or no EdU incorporation above background (Fig. 2*C*).

An S-phase arrest in response to EGFR inhibition is uncommon, as receptor signaling is typically required to bypass the G1/S “start” point of the cell cycle but is thought to no longer be required once DNA synthesis has begun (32). Mechanisms that do cause S-phase arrest typically arise from the DNA damage response (DDR), activated either by DNA damage or replicative stress (33, 34). The DDR activates p53 and/or checkpoint kinases (*e.g.*, Chk1 or Chk2) downstream of the DNA damage sensors (*e.g.*, ATM, ATR, and DNA-dependent protein kinase) that phosphorylate cell cycle regulatory factors, among them the histone variant H2AX (33, 34, 35). However, UM-SCC47 or MDA-MB-231 tumor cells arrested in response to SSTN_EGFR_ fail to activate either Chk1 or Chk2 or cause phosphorylation of H2AX (γH2AX) (Fig. S1). This contrasts with the phosphorylation observed when they are treated with ionizing radiation or hydroxyurea (HU) to induce DNA damage or replicative stress that leads to DDR activation (35) (Fig. S1).

The apparent failure of the SSTN_EGFR_-arrested cells to accumulate in any one phase of the cell cycle suggests that the block is global rather than focused on any one phase. To test this, we compared the behavior of UM-SCC47 cells subjected to either a double thymidine block or a double SSTN_EGFR_ block. The UM-SCC47 cells have a 24 h cell cycle, with an approximate 8 h S-phase, 2 h G2/M, and 14 h G1. Thus, EdU labels approximately one-third of the cells if they are not synchronized (Fig. 2*D*). A 24 h thymidine block arrests the one-third of the cells that are spread throughout S-phase, whereas the rest progress to the G1/S start before arresting. A subsequent 16 h release allows these and the cells arrested throughout S-phase to progress through and exit S-phase and enter G1. Reimposition of a second 24 h thymidine block at this point prevents these cells from entering S-phase and they arrest as a cohort at the G1/S interface. Cells labeled at this point show no EdU incorporation (0 h), but cells released for 4 or 6 h transit partially through S-phase, as shown by extensive (greater than 80%) EdU labeling (Fig. 2*D*). This contrasts with the result seen if the cells are subjected to a 24 h double SSTN_EGFR_ block (Fig. 2*D*). The cells show no labeling for 1 h after SSTN_EGFR_ removal, but at 2, 4, or 6 h after release, approximately one-third of the cells incorporate EdU, the same as cells treated with vehicle alone, indicating that the number of cells in S-phase at any of these time points remains the same (Fig. 2*D*). There is no increase in cells entering S-phase, suggesting that cells have not piled up at the G1/S-phase boundary despite the ability of SSTN_EGFR_ to arrest cells in S-phase (Fig. 2*C*) nor have cells entering S-phase been delayed by a block elsewhere. Instead, the cells appear to globally arrest wherever they are in the cell cycle when SSTN_EGFR_ is applied and then recover and resume their transit when SSTN_EGFR_ is removed.

### Cell cycle progression depends on active RON and ABL1 kinases

**Figure 3:**
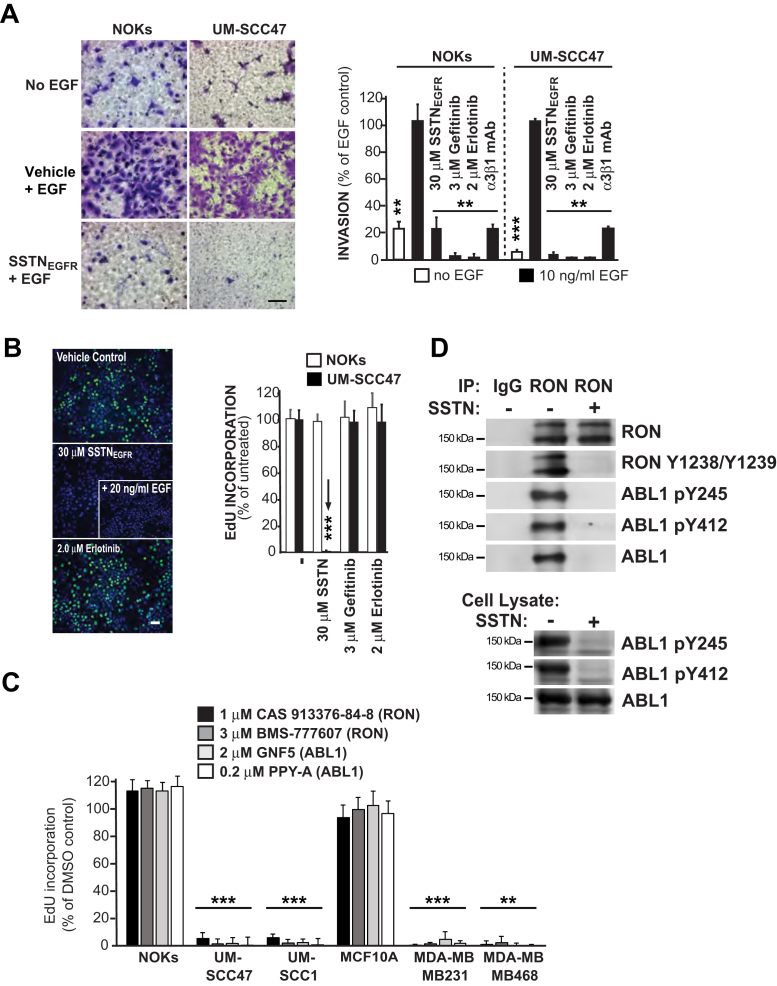
**Comparison of cell migration *versus* proliferation in the presence of SSTN**_**EGFR**_**, EGFR kinase inhibitors, or kinase inhibitors specific for RON or ABL1.***A*, NOK or UM-SCC47 HNSCC cells were induced to migrate across LN332-coated filters in the presence or absence of 10 ng/ml EGF or 10 ng/ml EGF together with 30 μM SSTN_EGFR_, 3 μM gefitinib, 2 μM erlotinib, or 10 μg/ml α3β1 blocking antibody (P1B5) (Bar = 50 μM). Migration is quantified and expressed as percent of migration relative to EGF-stimulated cells; *B*, UM-SCC47 HNSCC or NOKs grown in complete culture medium were treated for 3 h with 30 μM SSTN_EGFR_, 3 μM gefitinib, or 2 μM erlotinib, then fixed and stained with EdU and DAPI to quantify the percentage of cells synthesizing new DNA relative to control cells treated with vehicle alone. *Arrow* highlights the decreased proliferation of UM-SCC47 cells in SSTN_EGFR_. Representative pictures show untreated *versus* treated UM-SCC47 cells, including a cohort treated with 30 μM SSTN_EGFR_ supplemented with 10 ng/ml EGF (Bar = 50 μM); *C*, normal epithelial cells (NOKs, MCF10A), HNSCC cells (UM-SCC47, UM-SCC1), and TNBC cells (MB-468, MB-231) were grown for 3 h in complete culture medium containing RON kinase inhibitors (1 μM CAS 913376-84-8 or 3 μM BMS-77760l7) or ABL1 inhibitors (2 μM GNF5 or 0.2 μM PPY-A) followed by EdU labeling and quantification relative to control cells treated with vehicle alone; *D*, UM-SCC47 cells were treated for 3 h with vehicle or 30 μM SSTN_EGFR_, then lysed and the cell lysates were either (i) subjected to immunoprecipitation with nonspecific isotype control goat IgG or goat polyclonal anti-RON antibody (1 μg AF691/1 mg input) and analyzed on Western blots for total and active (pY1238/pY1239) RON and total and active (pY245 and pY412) ABL1 or (ii) analyzed on Western blots for active (pY245 or pY412) ABL1; ∗∗*p* ≤ 0.01, ∗∗∗*p* ≤ 0.001. EdU, 5-ethynyl-2′-deoxyuridine; EGF, epidermal growth factor; EGFR, epidermal growth factor receptor; HNSCC, head and neck squamous cell carcinoma; LN332, laminin-332; NOK, normal oral keratinocytes; RON, recepteur d’origine nantais; TNBC, triple-negative breast cancer.

We have shown previously that EGFR-stimulated migration of HaCaT and MCF10A epithelial cells on LN332 depends on its coupling with the α3β1 and α6β4 integrins by Sdc4 and requires EGF and active EGFR kinase (7, 13). Similarly, EGF-stimulated migration of NOKs or UM-SCC47 cells through LN332-coated filters is blocked by EGFR kinase inhibitors (gefitinib or erlotinib), SSTN_EGFR_, or α3β1 integrin blocking antibody (Fig. 3*A*). In contrast, the proliferation of NOKs or UM-SCC47 cells is independent of EGFR kinase activity, shown by the failure of gefitinib and erlotinib to block EdU incorporation even when used at 100-fold over their IC_50_ (Fig. 3*B*). But, consistent with the proliferation inhibition observed in Figure 1, the UM-SCC47 failed to incorporate EdU in the presence of SSTN_EGFR_ and the addition of EGF cannot reverse the block to EdU incorporation by SSTN_EGFR_ in the UM-SCC47 tumor cells (Fig. 3*B*).

These findings prompted us to question the potential role of other kinases in the arrest mechanism. Like EGFR (7, 13, 36), RON is reported to associate with the α6β4 integrin (37, 38), although whether this association is direct or mediated by a syndecan has not been investigated. Accordingly, we tested kinase inhibitors specific for RON (CAS 913376-84-8 and BMS-777607). We also tested GNF-5 and PPY-A, kinase inhibitors specific for ABL1, a cytoplasmic kinase known to be activated by RON (39, 40) and found that inhibiting either kinase mimics the effects of SSTN_EGFR_ by significantly reducing EdU incorporation in HNSCC and TNBC cells, but not in NOKs or MCF10A cells (Fig. 3*C*). Focusing on the UM-SCC47 cells, we find that RON and ABL1 are active in the tumor cells, as evidenced by phosphorylation of Y1238/1239 and Y412 in their respective kinase domains, as well as Y245 in the SH2-kinase linker of ABL1 (29, 41) (Fig. 3*D*). This phosphorylation is blocked by SSTN_EGFR_, and ABL1 no longer associates with RON (Fig. 3*D*). This suggests that by interacting with Sdc4, either directly or indirectly, RON is activated *via* transphosphorylation when clustered, engages the ABL1 SH2 domain, and activates ABL1 by phosphorylation.

**Figure 4:**
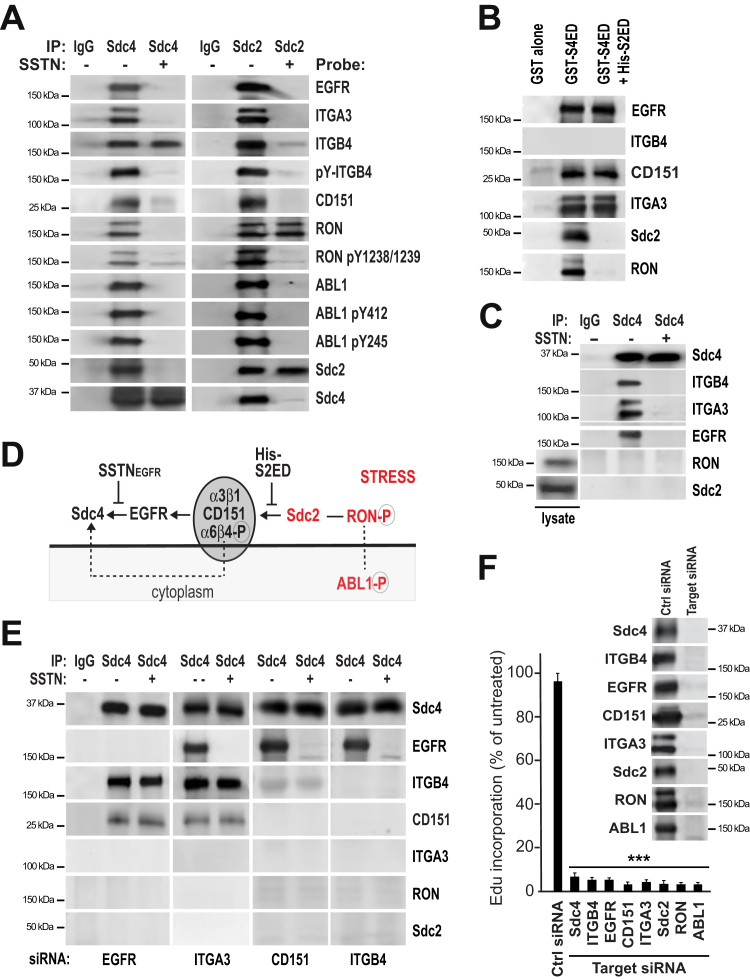
**Identification of signaling receptors required for S-phase progression.***A*, UM-SCC47 cells treated with vehicle alone or 30 μM SSTN_EGFR_ for 3 h were lysed and subjected to immunoprecipitation with nonspecific, isotype-matched control IgG, and mAb 8G3 to Sdc4 or rabbit polyclonal antibody to Sdc2. Immunoprecipitates were probed for the presence of EGFR, α3 integrin (ITGA3), β4 integrin (ITGB4), phosphorylated ITGB4 with PY20, CD151, total and active RON (pY1238/1239), total and active ABL1 (pY412 and pY245), and Sdc2 and Sdc4; *B*, GST-S4ED immobilized on glutathione beads was incubated with UM-SCC47 whole-cell lysates overnight in the presence or absence of 30 μM His-tagged S2ED and the receptors captured by S4ED were analyzed by Western blot; *C*, Sdc4 immunoprecipitates from HaCaT whole-cell lysates were probed for associated α3 integrin (ITGA3), β4 integrin (ITGB4), EGFR, RON, and Sdc2 *via* Western blot. RON and Sdc2 levels in the total lysate are shown. *D*, model showing proposed order of receptor complex assembly. *E*, UM-SCC47 cells transfected for 72 h with siRNA specific for human EGFR (3′UTR), α3 integrin (ITA3; s7543), CD151 (s194332), or β4 integrin (ITGB4; s7584) before performing Sdc4 immunoprecipitation and probing for associated receptors. Protein expression in starting cell lysates is shown in Fig. S2*A*. Results are representative of duplicate experiments with cells transfected with two different siRNA oligos for each targeted protein (see Fig. S2*B*); *F*, UM-SCC47 cells were treated for 72 h with either control siRNA (AM4635) or siRNA-specific anti-human Sdc4 (12434), β4 integrin (ITGB4; s7584), α3 integrin (ITGA3; s7543), EGFR (3′ UTR), CD151 (s194332), Sdc2 (s12635), RON (s8996), or ABL1 (s865), then labeled with EdU to quantify DNA synthesis; ∗∗∗*p* ≤ 0.001. Western blot inset shows individual receptor expression 72 h after siRNA transfection. Results are representative of duplicate experiments with cells transfected with two different siRNA oligos for each targeted protein. EGFR, epidermal growth factor receptor; RON, recepteur d’origine nantais; Sdc2, syndecan-2; Sdc4, syndecan-4.

Probing Sdc4 immunoprecipitates from UM-SCC47 cells confirms that active RON and active ABL1 coprecipitate with Sdc4 (Fig. 4*A*), along with EGFR, the α3β1 integrin, phosphorylated α6β4 integrin, the CD151 tetraspanin known to associate with the α3β1 and α6β4 integrins (42, 43, 44, 45, 46, 47, 48), and Sdc2–Sdc4 homolog (49). All are displaced by SSTN_EGFR_ with the exception of the α6β4 integrin, which remains engaged with Sdc4 *via* its cytoplasmic domain (13) but is no longer phosphorylated (Fig. 4*A*). This recapitulates prior findings that phosphorylation of the α6β4 integrin depends on kinases in the receptor complex that are activated by syndecan clustering (13, 36, 50). Immunoprecipitation of Sdc2 captures the same cohort of receptors (Fig. 4*A*), strongly suggesting that they are all in a single receptor complex. SSTN_EGFR_ displaces all but RON from Sdc2 (Fig. 4*A*), although RON is no longer phosphorylated, explaining the loss of ABL1 as well. This suggests that RON relies on Sdc2 for its linkage to the receptor complex, potentially *via* an interaction involving the Sdc2 extracellular domain. To confirm this, the receptors that assemble together by docking, either directly or indirectly, with the Sdc4 extracellular domain (namely, Sdc2, EGFR, CD151, α3β1 integrin, and RON) were captured using GST-S4ED, a recombinant Sdc4 extracellular domain fusion protein (Fig. 4*B*); performing this capture in the presence of recombinant His-tagged Sdc2 extracellular domain as a competitor prevents capture of Sdc2 and RON (Fig. 4*B*).

The incorporation of Sdc2, RON, and ABL1 into the receptor complex in tumor cells contrasts with the makeup of the complex in nontumorigenic cells; originally defined in HaCaT cells, Sdc4 immunoprecipitates contain EGFR and α6β4 and α3β1 integrins that the HaCaT cells use for migration on LN332 (7, 13). These precipitates do not contain Sdc2 and RON, although these receptors are expressed by the cells (Fig. 4*C*). These findings suggest that in tumor cells, Sdc2 becomes incorporated into the receptor complex involved in cell adhesion and migration and extends its activity to a mechanism that sustains cell proliferation as well (model Fig. 4*D*).

The lack of a requirement for EGFR kinase activity in the cell proliferation mechanism raises the question of what role, if any, EGFR has in this receptor complex. Silencing EGFR expression blocks assembly of the entire complex with Sdc4 except for the α6β4 integrin that remains bound *via* its cytoplasmic domain and CD151 that is known to associate with the integrin (Figs. 4*E* and S2*B*), revealing a major structural role for EGFR. Silencing α3β1 integrin expression does not affect the assembly of this core set of receptors, but it does prevent the capture of Sdc2 and Sdc2-associated RON kinase (Figs. 4*E* and S2*B*). Furthermore, silencing either CD151 or α6β4 integrin has no effect on EGFR engaging Sdc4 but does block the assembly of the adhesion receptor complex consisting of α3β1, α6β4, and CD151 with EGFR (Figs. 4*E* and S2*B*). Control siRNA has no effect on the assembly of the receptor complex (Fig. S2*B*) or expression of the complex’s component receptors (Fig. S2*A*). Moreover, each receptor siRNA is target-specific with no off-target effects on expression of other components within the complex (Fig. S2*A*). These findings provide a general order of assembly as shown in Figure 4*D*, with EGFR providing a link between Sdc4 and a complex of CD151 and the integrins and Sdc2 linking RON to this complex *via* an as-yet undefined interaction of the Sdc2 extracellular domain. Each member of the receptor complex appears to play an essential role either in assembly of the receptor complex or in signaling because silencing expression of any one of the receptors or kinases (*e.g.*, Sdc4, Sdc2, EGFR, RON, α3β1 integrin, α6β4 integrin, CD151, or ABL1) results in cessation of DNA synthesis by well over 90% in UM-SCC47 cells (Fig. 4*F*).

### Cell surface expression of cell cycle regulatory receptors is upregulated by carcinoma cells

**Figure 5:**
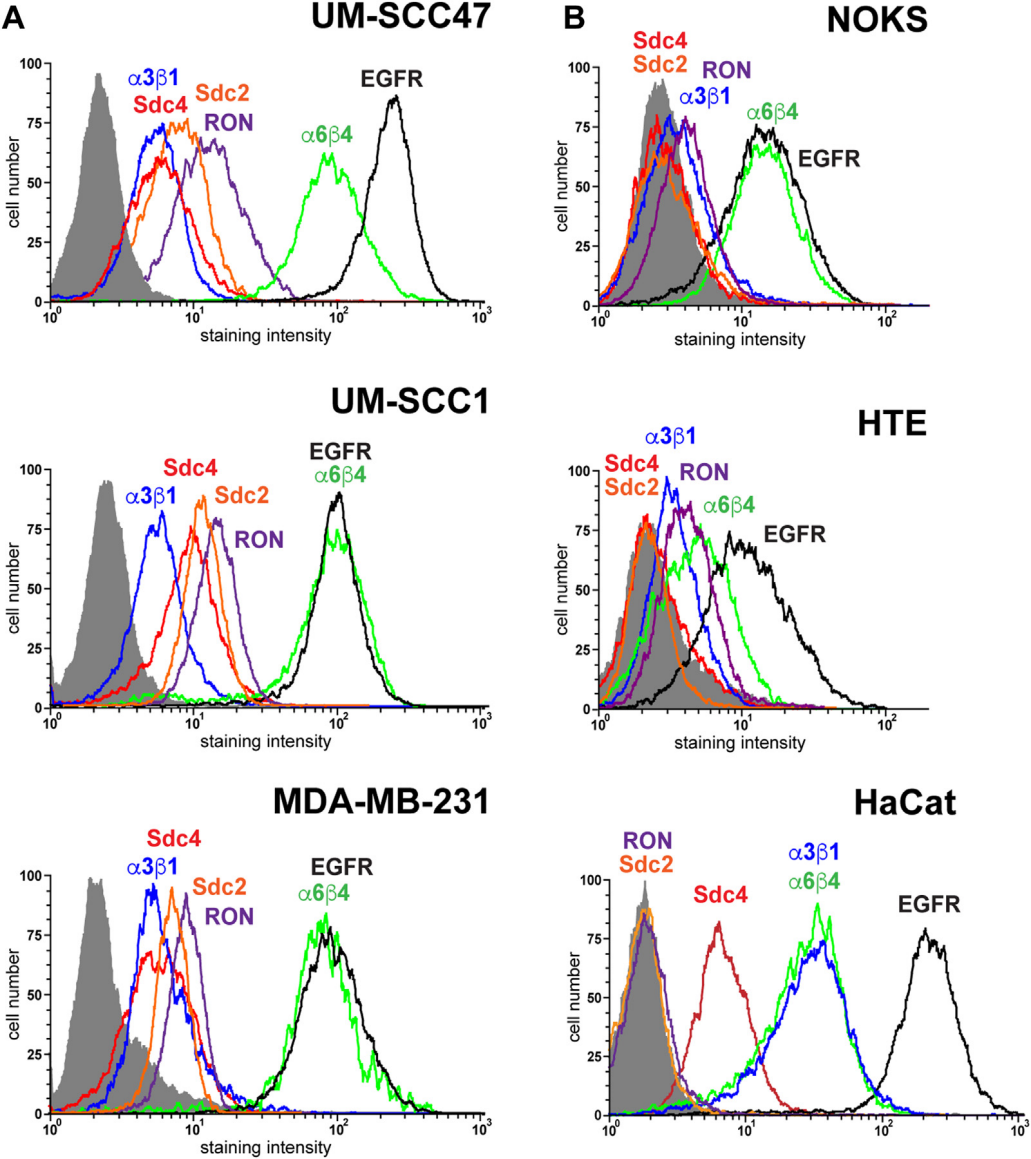
**Relative expression of members of the Sdc4:RTK:ITG complex on nontumorigenic and neoplastic epithelial cells.** Cell surface of expression of Sdc4 (mAb 8G3, *red*), Sdc2 (mAb 305515, *orange*), α3β1 (mAb P1B5, *blue*), α6β4 (mAb 3E1, *green*), EGFR (mAb EGFR.1, *black*), and RON (FAB6647F, *purple*) were analyzed by flow cytometry compared to nonspecific IgG (gray profile) on *A*, HNSCC (UM-SCC47, UM-SCC1) and TNBC (MDA-MB-231) cells or *B*, nontransformed HaCaT, HTE, or NOK epithelial cells. EGFR, epidermal growth factor receptor; HNSCC, head and neck squamous cell carcinoma; HTE, human tonsillar epithelial cell; NOK, normal oral keratinocyte; RON, recepteur d’origine nantais; Sdc2, syndecan-2; Sdc4, syndecan-4; TNBC, triple-negative breast cancer.

Analysis of receptor expression on representative carcinoma cell lines demonstrates that all express the critical signaling and adhesion receptors of the Sdc4-organized complex at the cell surface (Fig. 5*A*). This contrasts with nontransformed epithelial cells. HaCaT cells express cell surface Sdc4, EGFR, α3β1 integrin, and α6β4 integrin but have undetectable levels of cell surface RON and Sdc2 despite the presence of these receptors in HaCaT cell lysates (*cf.* Fig. 4*C*). NOK and HTE cells express EGFR and the integrins but have undetectable levels of the Sdc2 and RON, as well as Sdc4, at the cell surface (Fig. 5*B*).

### RON and ABL1 suppress activation of p38MAPK

**Figure 6:**
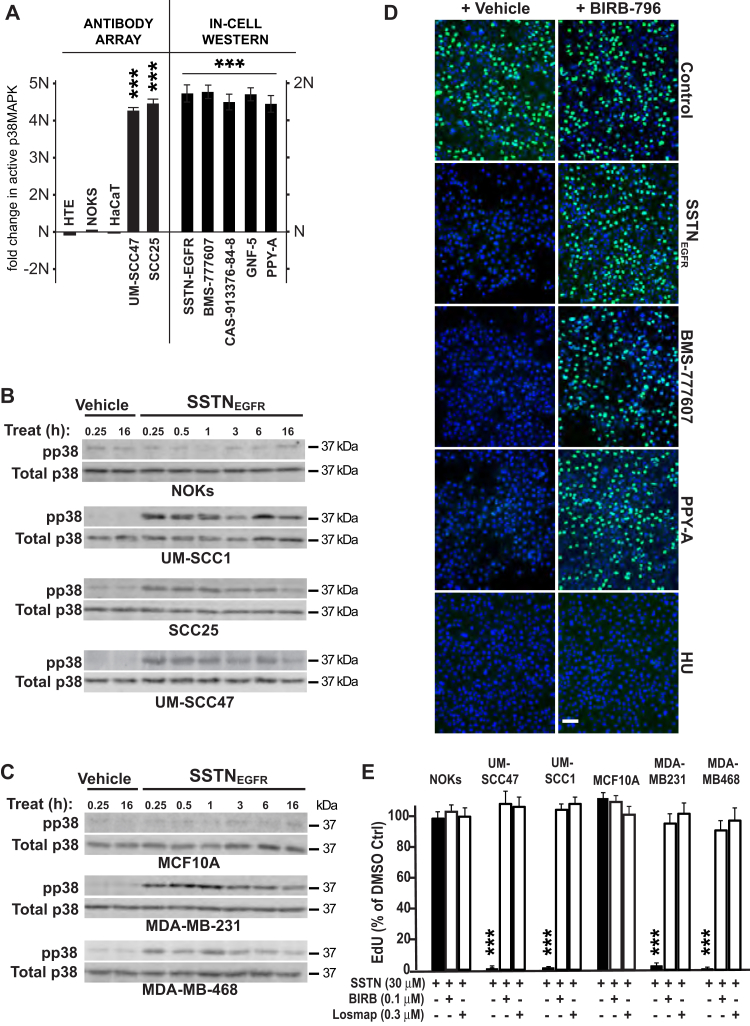
**SSTN-induced cell cycle arrest depends on activated p38MAPK.***A*, quantification of p38MAPK activation by antibody array (pT180/pY182) in nontransformed keratinocytes (HTE, NOK, and HaCaT) and HNSCC cells (UM-SCC47, SCC25) treated with 30 mM SSTN_EGFR_ (*left*) or in-cell western of UM-SCC47 cells following treatment with SSTNEGFR, RON kinase inhibitors (BMS-777607 or CAS-913376-84-8), or ABL1 inhibitors (GNF-5 or PPY-A)(*right*); *B*, detection of pT180/pY182 p38MAPK following treatment with vehicle alone or 30 μM SSTN_EGFR_ in nontransformed NOKs or transformed HNSCC cells; *C*, in nontransformed (MCF10A) or transformed (MDA-MB-231, MDA-MB-468, SKBr3) mammary epithelial cells; *D*, EdU-Click-IT–labeled UM-SCC47 cells treated for 3 h with either vehicle (control), 30 μM SSTN_EGFR_, 3 μM BMS-777607 (RON inhibitor), 0.2 μM PPY-A (ABL1 inhibitor), or 5 mM hydroxyurea (HU) in the presence or absence of 0.1 μM p38MAPK inhibitor BIRB-796 (Bar = 50 μm); *E*, quantification of EdU incorporation into cells treated with 30 μM SSTN_EGFR_ with or without p38MAPK inhibitors BIRB-796 or Losmapimod for 3 h; ∗∗∗*p* ≤ 0.001. EdU, 5-ethynyl-2′-deoxyuridine; HNSCC, head and neck squamous cell carcinoma; HTE, human tonsillar epithelial cell; NOK, normal oral keratinocyte; P38MAPK, p38 mitogen-activated protein kinase.

In the course of these studies, we probed an antibody array looking for signaling markers that change in SSTN_EGFR_-treated tumor cells but not normal epithelial cells. This revealed an upregulation of stress-activated p38MAPK (Fig. 6*A*) in the tumor cells treated with SSTN_EGFR_ for 24 h. A time course of SSTN_EGFR_ treatment from 15 min to 16 h confirms that HNSCC and TNBC cells all activate p38MAPK within 15 min of peptide addition and that activation persists throughout the entire time course examined, whereas NOKs and MCF10A cells fail to activate the kinase (Fig. 6, *B* and *C*). To test if activated p38MAPK is responsible for the S-phase arrest observed when SSTN_EGFR_ causes inactivation of RON and ABL1, UM-SCC47 cells were treated with either SSTN_EGFR_, RON kinase inhibitor (BMS-777607 or CAS 913376-84-8), or ABL1 kinase inhibitor (PPY-A or GNF-5) for 3 h in the presence or absence of p38MAPK inhibitor BIRB-796 (doramapimod). Treatment with either RON or ABL1 kinase inhibitor alone activates p38MAPK (Fig. 6*A*, In-Cell Western) and blocks EdU incorporation (Fig. 6*D*), which is fully rescued by the addition of BIRB-796 (Fig. 6, *A* and *D*). Arrest caused by activating the DDR using HU is not reversed by BIRB-796 (Fig. 6*D*). This reversal extends to each of the representative HNSCC and TNBC cell lines treated with SSTN_EGFR_, all of which are rescued (Fig. 6*E*) either with BIRB-796 or another p38MAPK inhibitor, losmapimod (also known as GW856553X, SB856553, or GSK-AHAB).

## Discussion

We have defined a novel signaling apparatus organized by Sdc4 that suppresses cell cycle arrest in carcinoma cells, thus allowing their continued proliferation. Disruption of the Sdc4-organized signaling mechanism using a competitor SSTN_EGFR_ peptide leads to a rapid and global arrest of the carcinoma cells throughout the cell cycle. The signaling mechanism builds on a previously described receptor complex found on migrating epithelial cells comprised of the α3β1 integrin and EGFR docked to a juxtamembrane site in the Sdc4 ectodomain and the α6β4 integrin engaged by the Sdc4 cytoplasmic domain (7, 13). Whereas the cells rely on active EGFR kinase within this receptor complex to drive cell migration (36, 50), the proliferation regulatory mechanism described here is independent of EGFR kinase and depends on the incorporation of additional components into the complex, namely, Sdc2, RON, and the RON-associated kinase ABL1. These components appear to be critical for suppressing p38MAPK activity in order to sustain the proliferation of transformed epithelial cells. Although not examined in this study, it is likely that this mechanism is a normal response of epithelial cells to changes in their environment, perhaps coupled to stress signals encountered during migration and wound healing. For example, suspension of epidermal keratinocytes activates p38MAPK, which is suppressed if the cells are allowed to readhere to LN332 *via* the α3β1 and α6β4 integrins (51).

**Figure 7:**
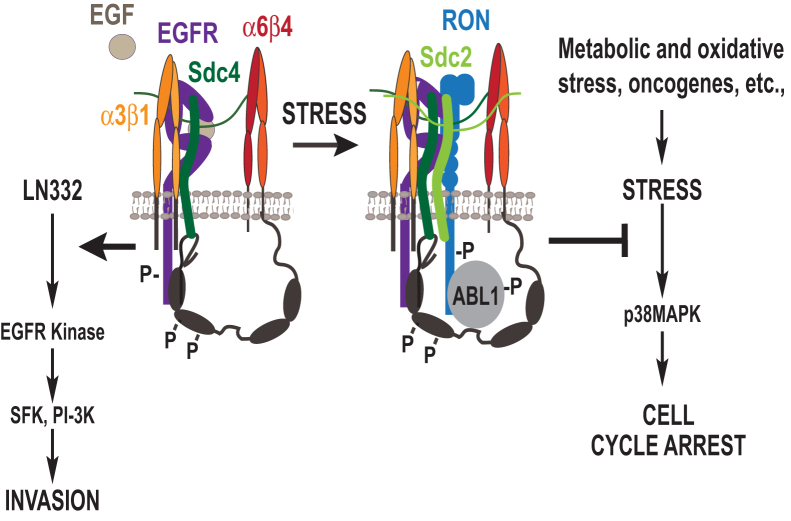
**Model.** Model showing the role of the syndecan-organized multi-receptor complex in promoting EGFR kinase–dependent cell invasion and EGFR kinase–independent suppression of stress-induced S-phase arrest in tumor cells. EGFR, epidermal growth factor receptor.

By suppressing p38MAPK, the syndecan-organized receptor complex may ensure continued DNA synthesis in the tumor cells that are undergoing oncogenic, metabolic, or genotoxic stress (see model, Fig. 7). Increasing evidence suggests that p38MAPK and c-Jun N-terminal kinase stress kinases have significant roles in cell cycle arrest, whether caused by DNA damage or other types of stress (52, 53, 54). Although their targets are only now being identified, p38MAPK is known to target p38MAPK-activated protein kinase-2 (MAPK-APK2) that inactivates the cdc25A phosphatase and causes G1- and S-phase arrest (55). Interestingly, prior studies have shown that proliferation of prostate and breast carcinoma cells is enhanced by RON activation of ABL1, which is proposed to phosphorylate tyrosine 211 in proliferating cell nuclear antigen (PCNA), the sliding clamp that assembles the replisome engaged in DNA synthesis (39, 40, 56). Disruption of this event by SSTN_EGFR_ may also have a role in the overall arrest mechanism, although how p38MAPK would be involved in this mechanism is not clear.

How these newly identified receptors are incorporated into the receptor complex remains unknown, but our current data provide a plausible order of assembly (Fig. 4*D*). The α6β4 integrin binds a motif in the C-terminus of the Sdc4 cytoplasmic domain and there is no evidence to date for an extracellular interaction between this integrin and the syndecan (13, 57). But all of the remaining receptors are displaced from Sdc4 by SSTN_EGFR_, indicating that they all rely either directly or indirectly on the docking site comprised by amino acids 87 to 131 in the Sdc4 extracellular domain. Sdc4’s interaction with EGFR is direct, as it is known that purified recombinant EGFR and Sdc4 extracellular domains interact directly *via* this site (7). EGFR, in turn, may recruit the α3β1 integrin contained in a subcomplex consisting of the α3β1 and α6β4 integrins and the tetraspanin CD151 (Fig. 4*D*), most likely in a specialized, tetraspanin-enriched membrane microdomain (58, 59). Other examples where integrins and kinases are coupled by docking sites in syndecan extracellular domains (*e.g.*, α3β1 and HER2 (7), α4β1 and VEGFR2 (6), αvβ3 and IGF1R (5)) all involve some recognition of the syndecan by both the integrin and the kinase, which suggests α3β1 may depend on a bipartite interaction with EGFR and Sdc4.

Our current evidence strongly suggests that ABL1 engages the cytoplasmic domain of activated RON, which in turn engages a site in the extracellular domain of Sdc2. What remains unknown is the interaction that links Sdc2 to amino acids 87 to 131 in the extracellular domain of Sdc4. The most likely scenario is that Sdc2 also interacts with the α3β1 integrin, perhaps stabilized by an interaction between the α3β1 integrin and RON. This would explain why Sdc2 and its associated kinases are lost from Sdc4 when α3β1 integrin expression is silenced (Fig. 4*D*). There are likely to be other stabilizing interactions as well. Sdc2 and Sdc4 are known to heterodimerize *via* interactions within their transmembrane domains (60). RON may also form heterodimers with EGFR (61, 62), potentially further stabilizing the incorporation of Sdc2, RON, and ABL1 into the receptor complex.

RON is activated when incorporated into the receptor complex, presumably by clustering, leading to phosphorylation of ABL1 and the α6β4 integrin. This mimics the activation of HER2, IGF1R, and VEGFR2 when they become engaged with syndecans (5, 6, 7). The exception appears to be EGFR, which requires EGF ligand rather than clustering for activation when associated with Sdc4 and integrins (13). Future studies will be required to identify the target of ABL1 that restricts p38MAPK activation, presumably an upstream enzyme in the MAPK signaling cascade, as well as the target of p38MAPK that causes the global arrest.

## Experimental procedures

### Reagents

SSTN_EGFR_ peptide consists of amino acids 87 to 131 of human Sdc4 and was from LifeTein LLC. Antibodies include anti-human Sdc4 (F94-8G3) provided by Dr Guido David (University of Leuven) and integrin α6β4 (3E1) from Memorial Sloan-Kettering. Anti-Sdc4 (AF2918), EGFR (AF231), RON (AF691 and FAB6647F), pY1238/1239 RON (AF1947), ITGB4 (mAb 422325) Sdc2 (305515) were from R&D Systems . Anti-ITGA3 (NBP2-48514) was from Novus Biologicals. Rabbit mAbs 73E5 (pY245-ABL1), 247C7 (pY412-ABL1), D13E1 (p38MAPK), 11H10 (tubulin), 133D3 (pS345-CHK1), 2661S (pT68-CHK2), D3H8P (PCNA), 1B1B2 (histone H3), 28B10 (pT183/Y185-p38MAPK), and E4I9J (CD151) were from Cell Signaling Technology. Anti-MCM2 (A300-191A) was from Bethyl Laboratories. 8E9 (ABL1), TU-01 (tubulin), and 36-6200 (Sdc2) were from Invitrogen/ThermoFisher Scientific. DO-1 (p53) and H-7 (Sdc2) were from Santa Cruz Biotechnology. JBW301 (pS139-γH2AX), AC-74 (β-actin), and P1B5 (integrin α3β1) were from MilliporeSigma. Antibodies to Sdc2 were produced against GST-S2ED in rabbits and affinity-purified as described (63).

Erlotinib and gefitinib, BMS-0777607, BIRB-796 (Doramapimod), and Losmapimod were from Selleck Chemicals. Cetuximab was provided by Dr Paul Harari (University of Wisconsin-Madison). CAS 913376-84-8, actinomycin D, HU, and propidium iodide were from MilliporeSigma; GNF5 and PPY-A are from R&D Systems. EdU and Click-IT EdU-labeling reagents were from Click Chemistry Tools and MilliporeSigma. CellTiter-GLO was from Promega.

### Cell culture

Parental telomerase reverse transcriptase–immortalized human NOKs and HTEs were described previously (64, 65). HaCaT keratinocytes (CVCL 0038) were provided by Dr Peter LaCelle (Roberts Wesleyan College). Human mammary MCF10A (CVCL 0598), MDA-MB-231 (CVCL 0062), MDA-MB-468 (CVCL 0419) cells, and human SCC25 HNC (CVCL 1682) cells were from ATCC. UM-SCC47 (CVCL 7759), UM-SCC1 (CVCL 7707), TU-138 (CVCL 4910), and 93-VU-147T (CVCL L895) HNC cells were provided through the auspices of the Wisconsin Head and Neck Cancer SPORE. All cells with the exception of the NOKS and HTEs were STR profiled by Genetica LabCorp within 6 months of use. Cells were cultured at 37 °C and 92.5% air/7.5% CO_2_. NOKs and HTEs were cultured in complete Keratinocyte Serum-Free medium containing 100 units/ml penicillin and 100 μg/ml streptomycin (Life Technologies). All other cell lines were cultured as previously described (5, 7, 8, 9). New cultures were reestablished from frozen stocks after a maximum of 3 to 4 months of passage, and all cultures were screened for *mycoplasma* approximately every 6 months by the Small Molecule Screening Facility in the University of Wisconsin Carbone Cancer Center using the R&D Systems MycoProbe *Mycoplasma* Detection Kit (Cat. # CUL001B).

### Flow cytometry

To measure cell surface receptor expression, suspended cells were incubated for 1 h on ice with 1 μg of primary antibody per 5 × 10^5^ cells, washed, counterstained with Alexa-488-conjugated goat secondary antibodies, and scanned on a Thermo Fisher Scientific’s Attune NxT bench top cytometer. Cell scatter and PI staining profiles were used to gate live, single-cell events. For cell cycle analysis, asynchronous or synchronous (double thymidine block: 4 mM thymidine for 24 h, followed by 16 h release in culture medium supplemented with 30uM deoxycytidine (D3897, MilliporeSigma), followed by an additional 24 h with 4 mM thymidine) were released from the double thymidine block and treated with SSTN_EGFR_ after cells recovered from the block and entered S-phase. Cells were labeled with 100 μM EdU for 1 h prior to being suspended and fixed in ice-cold 70% ethanol. Cells were first stained using a Click-IT EdU-labeling reaction for 1 h (1.3 mM THPTA/CuSO_4_ mix, 20 μM AF488 picolyl azide, and 2.5 mM ascorbic acid in 0.1% Triton X-100) followed by DAPI (D1306) staining for 4 h (5 μg/ml in the presence of 1 μg/ml DNAse-free RNase A (Thermo Fisher Scientific) in 0.1% Triton X-100). Cells were then analyzed by flow cytometry to assess levels of AF488-EdU and DAPI staining on a ThermoFisher Attune NxT bench top cytometer.

### Immunoprecipitations and Western blotting

Immunoprecipitation of the Sdc–receptor tyrosine kinase: ITG complex in the presence or absence of competing SSTN_EGFR_ peptide was carried out using antibodies to Sdc4 or Sdc2 mAb (or mouse IgG1 as a negative control), GammaBind PLUS Sepharose (GE Healthcare Life Sciences), and 1 mg of precleared whole cell lysate (WCL)/sample in the presence of protease and phosphatase inhibitors as previously described (8, 9). For p38MAPK and DDR effector blots, equal amounts of total protein (20–40 μg depending on the target protein and cell line being probed) were loaded per lane. All samples were resolved on 10% Laemmli gels prior to transfer to Immobilon-FL PVDF (MilliporeSigma). Visualization of immunoreactive bands was performed using ECF reagent or direct excitation of fluorescent secondary antibodies on either a GE Healthcare Life Sciences Typhoon Trio or a LI-COR Odyssey Fc Imaging System. For the in-cell western of active p38MAPK, cells were fixed (4% paraformaldehyde), permeabilized (0.3% TX-100), blocked (5% normal goat serum), and then stained for phosphorylated (28B10) and total (D13E1) p38MAPK followed by IR secondary (Li-COR) 800CW for phosphorylated and 680RD for total p38MAPK. Cells were then scanned on a Li-COR Odyssey Aerius flat-top scanner and each cohort was quantitated for the ratio of phosphorylated to total p38MAPK as the fold change (minus background isotype-matched, nonspecific IgG staining) relative to vehicle-treated, control cells.

### siRNA design and transfection

Two different siRNAs were used for all treatments with equivalent results, paired with a scrambled siRNA control: *Silencer Select* control (AM4635) and target-specific siRNA oligos directed against human Sdc4 (siRNA ID# 12434, Target Sequence: ^199^(ca)GGAATCTGATGACTTTGAG^217^ and siRNA ID# s12638, Target Sequence: ^536^CTACTGCTCATGTACCGTA(tt)^554^; GenBank Accession number NM_002999.4), Sdc2 (siRNA ID# s12635, Target Sequence ^996^TGACCTTGGAGAACGCAAA(tt)^1014^ and siRNA ID# 12636 ^868^GACAGTCTGTTTAAACGGA(tt)^886^; GenBank Accession Number NM_002998.4), ITGB4 (siRNA ID# s7584, Target Sequence: ^658^GCGACTACACTATTGGATT(tt)^676^ and siRNA ID# s7585, Target Sequence: ^580^CCAACTCCATGTCCGATGA(tt)^598^; GenBank Accession Number NM_001005731.3), ITGA3 (siRNA ID# s7543, Target Sequence: ^1026^GGACTTATCTGAGTATAGT(tt)^1044^ and siRNA ID# s7541, Target Sequence: ^2629^GTAAATCACCGGCTACAAA(tt)^2647^;GenBank Accession Number NM_002204.4), EGFR (3′UTR Target Sequence: ^4905^TGCTCTGAAATCTCCTTTAtt^4923^, GenBank Accession Number NM_005228.5) and human EGFR-specific siRNA oligo (sc-29301) acquired from Santa Cruz Biotechnology, MST1R (siRNA ID# s8996, Target Sequence: ^3576^GGCCCAGAATCGAATCCAA(tt)^3594^ and siRNA ID# s8998, Target Sequence: ^3065^GCGTAGATGGTGAATGTCA(tt)^3084^; GenBank Accession Number, NM_002447.4), ABL1 (siRNA ID# s865, Target Sequence ^2030^CGACAAGTGGGAGATGGAA(tt)^2048^ and siRNA ID# s864, Target Sequence: ^1836^GAAGGGAGGGTGTACCATT(tt)^1854^; GenBank Accession Number NM_007313.3), and CD151 (siRNA ID# s2728, Target Sequence: ^409^CTGCTGCGCCTGTACTTCA(tt)^428^ and siRNA ID# s194332, 3′UTR Target Sequence: ^935^CCCAACTACTGAGCTGAGA(tt)^953^; GenBank Accession Number NM_004357.5)are from Life Technologies. UM-SCC47 cells (0.35 × 10^6^ per 35 mm well) were transfected with 100 nM siRNA using Lipofectamine RNAiMAX and Opti-MEM I transfection medium (from Life Technologies) at 1:1 ratio (μg siRNA:μl RNAiMAX). At 6 h post-transfection, the wells were supplemented with 10% fetal bovine serum and 3 ml of complete culture medium. At 24 h post-transfection, the cells were suspended and plated on either acid-etched 18mm-#1 glass coverslips in 12-well plates or in 60 mm tissue culture plates. Cells were then harvested at 72 h post-transfection and analyzed by Western blot (for receptor expression levels loading equal cell equivalents per sample) or AF488-EdU staining (for active DNA synthesis).

### Immunofluorescence

Cells were plated on glass coverslips overnight and then treated with or without SSTN_EGFR_ peptide in the presence or absence of the indicated inhibitors for the times indicated. For EdU labeling, cells were incubated with 100 μM EdU 45 min prior to fixation. Cells were fixed in 4% paraformaldehyde, permeabilized in 0.5% Triton X-100 in 1× CMF-PBS (pH 7.4), and blocked for 1 h at RT in a 3% bovine serum albumin (BSA)/CMF-PBS solution. The cells were then stained using a Click-IT EdU-labeling reaction for 30 min at RT (1.3 mM THPTA/CuSO_4_ mix, 20 μM AF488 picolyl azide and 2.5 mM ascorbic acid in a 1% BSA/CMF-PBS solution) prior to washing with PBT solution (CMF-PBS containing 1% BSA and 0.2% Tween-20) and mounting in ProLong Diamond Antifade Mountant with DAPI (Life Technologies). For PCNA staining, cells were first hypotonically lysed in a buffer containing 10 mM Tris (pH 7.4), 2.5 mM MgCl_2_, 0.5% NP-40, HALT protease and phosphatase inhibitor cocktail, and 1 mM DTT for 15 min under constant agitation. The cells were then washed with 1× CMF-PBS (pH 7.4), then blocked in a 3% BSA/CMF-PBS solution for 1 h prior to Click-IT EdU AF488-labeling, as described previously. Cells were then stained mAb D3H8P (1:800) for 1.5 h followed by Alexa546-conjugated secondary antibody prior to mounting. Fluorescent images (six representative fields from duplicate wells/experiment) were acquired using either a Zeiss PlanAPOCHROMAT 10× (0.45 NA) or 20× objective (0.8 NA) and a Zeiss AxioCam Mrm CCD camera on a Zeiss Axio Imager.M2 microscopy system. Stained cells were quantified using ImageJ (https://imagej.nih.gov/ij/) using threshold limits based on controls.

### Cell proliferation assays

Cells (1–2 × 10^3^/well) were plated in 96-well plates in complete culture medium in the presence or absence of SSTN_EGFR_ peptide or EGFR kinase inhibitors for 72 h. Cell numbers were measured using CellTiter-GLO against a standard curve in accordance with the manufacturer’s instructions.

### Cell stress and apoptosis marker array

Screening for stress markers activated in cells treated with SSTN_EGF1R_ peptide for 24 h relative to vehicle-treated (50 mM Hepes, pH 7.4) control cells was conducted as previously described (9) using the PathScan Stress and Apoptosis Signaling Antibody Array Kit (Cell Signaling Technology).

### Statistical analyses

Statistical analyses using a two-tailed Student’s *t* test were performed using Excel (Microsoft Office 365). Data that satisfy confidence levels of *p* ≤ 0.05, 0.01, or 0.001 are noted. Data are presented as means ± SEM, unless otherwise noted.

## Data availability

All data described in the article are contained herein.

## Supporting information

This article contains supporting information.

## Conflict of interest

The authors declare that they have no conflicts of interest with the contents of this article.

## Supporting information

Supplemental Figure S1 Analysis of DDR markers during SSTN_EGFR_ treatment. UM-SCC47 or MDA-MB-231 cells were treated with vehicle alone for 6 h, 30 μM SSTN_EGFR_ for either 1 h or 6 h, or 8 Gy ionizing radiation (IR) or 5 mM hydroxyurea (HU) for 1 h before being analyzed for activation of DNA damage response effectors using mAbs to pS345-Chk1, pT68-Chk2, or pS139-H2AX. Tubulin and actin serve as loading controls

Supplemental Figure S2 Analysis of control *versus* target-specific siRNAs. *A*, Western blot of whole-cell lysates of UM-SCC47 cells transfected for 72 h with siRNAs used in Figure 4: control siRNA (AM4635) or siRNA specific for human EGFR (3′UTR), α3 integrin (ITA3; s7543), CD151 (s194332), or β4 integrin (ITGB4; s7584); *B*, UM-SCC47 cells transfected for 72 h with siRNAs before performing Sdc4 immunoprecipitation and probing for associated receptors (duplicate of experiment shown in Fig. 4*E* using an alternate set of siRNAs): siRNA specific for human EGFR (sc-29301), α3 integrin (ITA3; s7541), CD151 (s2728), or β4 integrin (ITGB4; s7585)
