# Supplementary figures and images for: Plasma membrane proteoglycans syndecan-2 and syndecan-4 engage with EGFR and RON kinase to sustain carcinoma cell cycle progression
Source: J Biol Chem. 2022 May 13;298(6):102029. doi: 10.1016/j.jbc.2022.102029 (PMC9190016; doi:10.1016/j.jbc.2022.102029)

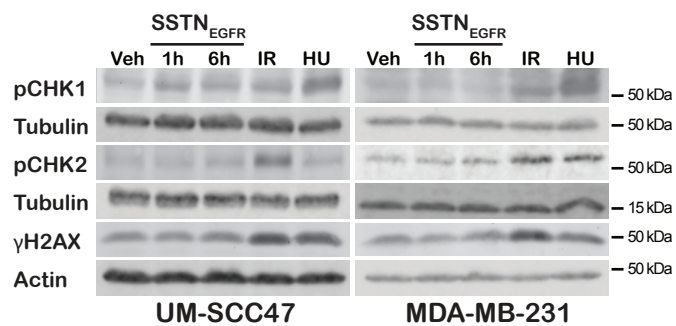

Supporting Information  
FIGURE 1S. BEAUVAIS ET AL.

Supplement: Supplemental Figure S1 — Analysis of DDR markers during SSTNEGFR treatment. UM-SCC47 or MDA-MB-231 cells were treated with vehicle alone for 6 h, 30 μM SSTNEGFR for either 1 h or 6 h, or 8 Gy ionizing radiation (IR) or 5 mM hydroxyurea (HU) for 1 h before being analyzed for activation of DNA damage response effectors using mAbs to pS345-Chk1, pT68-Chk2, or pS139-H2AX. Tubulin and actin serve as loading controls [file mmc1.pdf]

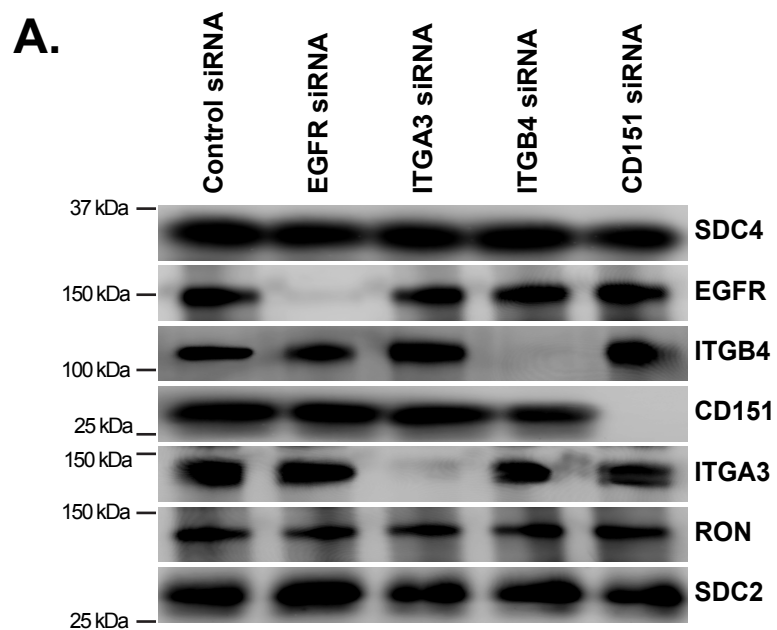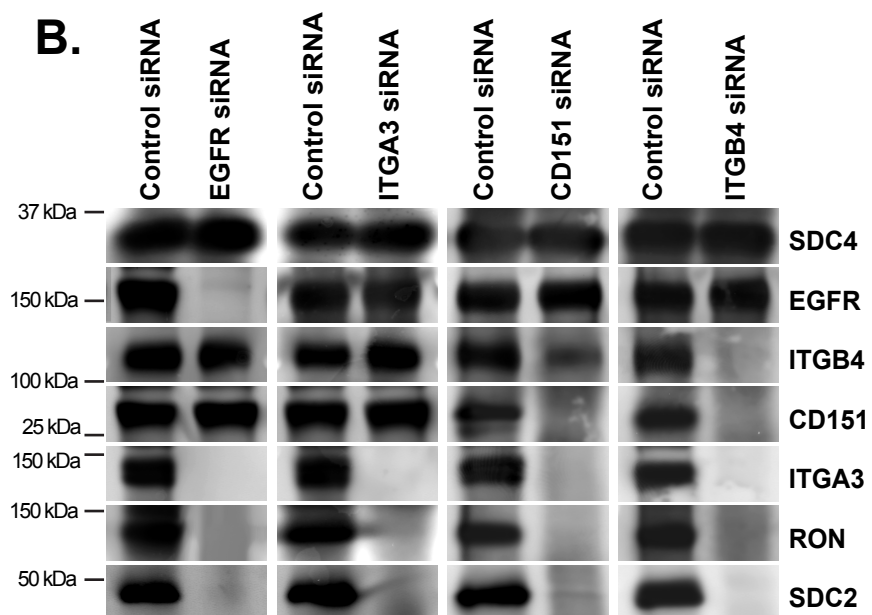

Supporting Information  
FIGURE S2. BEAUVAIS ET AL.

Supplement: Supplemental Figure S2 — Analysis of control versus target-specific siRNAs. A, Western blot of whole-cell lysates of UM-SCC47 cells transfected for 72 h with siRNAs used in Figure 4: control siRNA (AM4635) or siRNA specific for human EGFR (3′UTR), α3 integrin (ITA3; s7543), CD151 (s194332), or β4 integrin (ITGB4; s7584); B, UM-SCC47 cells transfected for 72 h with siRNAs before performing Sdc4 immunoprecipitation and probing for associated receptors (duplicate of experiment shown in Fig. 4E using an alternate set of siRNAs): siRNA specific for human EGFR (sc-29301), α3 integrin (ITA3; s7541), CD151 (s2728), or β4 integrin (ITGB4; s7585) [file mmc2.pdf]
